# Literature review to characterize the empirical basis for response scale selection in pediatric populations

**DOI:** 10.1186/s41687-018-0051-8

**Published:** 2018-09-06

**Authors:** April N. Naegeli, Jennifer Hanlon, Katharine S. Gries, Shima Safikhani, Anna Ryden, Mira Patel, Mabel Crescioni, Margaret Vernon

**Affiliations:** 1 Eli Lilly and Company, Lilly Corporate Center, DC1542, Indianapolis, IN 46285 USA; 2 Ironwood Pharmaceuticals, 301 Binney St, Cambridge, MA 02142 USA; 3 Janssen Global Services LLC, 700 US, 202 Raritan Ave, Raritan, NJ 08869 USA; 4 Evidera, 7101 Wisconsin Ave., Suite 1400, Bethesda, MD 20814 USA; 5 AstraZeneca Gothenburg, Pepparedsleden 1, 431 50 MöIndal, Sweden; 6 Critical Path Institute, 1730 E River Rd, Tucson, AZ 85718 USA

**Keywords:** Pediatric, Patient-reported outcome, Response option, Rating scale, Response scales, Children

## Abstract

**Background:**

Despite the importance of response option selection for patient-reported outcome measures, there seems to be little empirical evidence for the selected scale type. This article provides an overview of the published research on response scale types and empirical support within pediatric populations.

**Methods:**

A comprehensive review of the scientific literature was conducted to identify response scale option types appropriate for use in pediatric populations and to review and summarize the available empirical evidence for each scale type.

**Results:**

Eleven review/consensus guideline/expert opinion articles and 20 empirical articles that provided guidance or evidence regarding pediatric response scale selection were identified. There was general consensus that 5-point verbal rating scales, including Likert scales, were appropriate for children aged 7 or 8 and older, while graphical or faces scales are often used in pediatric studies with children of younger ages.

**Conclusion:**

In general, the verbal rating scale, numeric rating scale, visual analogue scale, and graphical scales have each demonstrated to be reliable and valid response option formats in specific contexts among pediatric populations; however, their appropriateness is dependent upon sample age. When selecting response scales, it is important to consider target population and context of use during the development of patient-reported outcome measures, especially with respect to tense, recall period, attribution, number of options, etc. In addition to age, cognitive development is an important aspect to consider for optimizing pediatric self-reported measures. More research is needed to determine clinically relevant changes and differences within pediatric research, which includes different response scale options.

## Background

The development of patient-reported outcome (PRO) measures involves the identification of the relevant concepts which are measured through one or more items (questions or statements (*items*)) that can be evaluated by utilizing a response option set. The response options must be consistent with each item’s purpose and intended usage. The selection of response options is an important component of item construction and characterizes how the concept is measured. When determining the type of response options to be used, many factors must be taken into account, most importantly the target population and intended use of the item. For instance, Lukas et al. [1] was able to demonstrate reliable reports of pain through appropriate selection of response types based upon the cognitive ability of the target population.

Historical use of response option types, the use of qualitative research and the assessment of measurement properties contribute to the identification of response categories that will perform most reliably within the intended population. Special consideration should be given to different populations, particularly the pediatric population. Important characteristics of pediatric populations influencing response set choice include age, literacy skills, ability to verbally communicate, cognitive ability to quantify feelings or symptoms, and motivational desire to please or select the ‘right’ answer [2, 3]. Therefore the development of a reliable and valid PRO measure for the pediatric patient population presents unique challenges.

The thoughtful selection of response options for new pediatric PRO measures is important, however, there is very little empirical basis for the type of response scale selected or attributes of the response scale including, number of response options, visual orientation of the scale (for example, vertical vs. horizontal), and response scale anchor wording. The United States Food and Drug Administration emphasizes the importance of self-report in pediatric populations rather than proxy-report, but do not recommend any specific response options for inclusion in a PRO measure. However, several response option types commonly seen in PRO instruments are listed for consideration in their 2009 Guidance for Industry, titled Patient-Reported Outcome Measures: Use in Medical Product Development to Support Labeling Claims [4]. Typical response options employed in PRO instruments include verbal rating scales (VRS), visual analogue scales (VAS), numeric rating scales (NRS), and various graphical scales such as a *Faces* scale. In the pediatric literature, it has been reported that children can reliably distinguish and understand fewer response options than adults; for example, in testing the Childhood Asthma Control Test, Liu et al. [5] found that a 4-point response scale was optimal. Further, graphical rather than numeric or verbal response scales may enhance comprehension of response scales in children [2].

The purpose of this article is to provide an overview of the published research on response scale option types used within pediatric populations. Evidence identified through this comprehensive literature review is intended to inform and enhance response scale selection for newly developed PRO instruments designed for use in pediatric populations.

## Methods

### Search procedure

A comprehensive review of the scientific literature was conducted to identify appropriate response scale option types for the pediatric population, and to review and summarize the available empirical evidence for each type of scale. The literature review was part of a larger study, funded by the Critical Path Institute’s Patient-Reported Outcome (PRO) Consortium, to summarize the available empirical evidence to support response option selection for PRO measures, by context of use. Published articles, limited to English-based in the preceding 10 years (2004–2014), were retrieved and reviewed to provide information on optimal response options for PRO measures in the pediatric population.

The search databases included EMBASE, MEDLINE, and PsycINFO. In addition to formal searches, a number of supplementary sources were utilized to identify additional relevant articles for inclusion in the review. Further, the reference lists of articles identified from the formal and supplementary searches were also reviewed to identify additional articles to be included in the review.

Lastly, a search was conducted for presentation abstracts that were accepted during the past two years of the meetings/conferences of the International Society for Pharmacoeconomics and Outcomes Research (2013 and 2014) and the International Society for Quality of Life Research (2012 and 2013) meetings/conferences to identify any scientific disclosures prior to publication in the peer-reviewed literature.

### Search strategy

Search terms used to identify articles in EMBASE, MEDLINE, and PsycINFO that met the search objective and were applicable to the pediatric population are presented in Table 1. Articles that provided both direct and indirect evidence were included. Direct evidence was defined as evidence that provided a direct answer to the research question of interest; for example, direct evidence articles empirically compared the relative robustness or merits of two different response scale types within the same study/population. Indirect evidence was defined as relevant evidence that should be considered in the review and overall conclusions, but that did not directly answer the research question or hypothesis. Articles were excluded if they provided no direct or indirect evidence relevant to the search objectives, were not applicable to PRO development, or addressed an area not pre-specified for inclusion.

**Table 1:** Literature review search terms

No.	Type	Search Terms
#1	Response scale terms	‘response scale’:ab,ti OR ‘response scales’:ab,ti OR likert:ab,ti OR ‘likert scale’/exp. OR ‘visual analog scale’:ab,ti OR ‘visual analog scales’:ab,ti OR ‘visual analogue scale’:ab,ti OR ‘visual analog scale’/exp. OR ‘numerical rating scale’:ab,ti OR ‘numerical rating scales’:ab,ti OR ‘verbal rating scale’:ab,ti OR ‘verbal rating scales’:ab,ti OR ‘competence scale’:ab,ti OR ‘competence scales’:ab,ti OR ‘frequency scale’:ab,ti OR ‘frequency scales’:ab,ti OR ‘extent scale’:ab,ti OR ‘extent scales’:ab,ti OR ‘comparison scale’:ab,ti OR ‘comparison scales’:ab,ti OR ‘performance scale’:ab,ti OR ‘performance scales’:ab,ti OR ‘developmental scale’:ab,ti OR ‘developmental scales’:ab,ti OR ‘qualitative scale’:ab,ti OR ‘qualitative scales’:ab,ti OR ‘agreement scale’:ab,ti OR ‘agreement scales’:ab,ti OR ‘categorical scale’:ab,ti OR ‘categorical scales’:ab,ti
#2	Selecting terms	select*:ab,ti OR choos*:ab,ti OR criteria:ab,ti OR compare:ab,ti OR comparison:ab,ti
#3	PRO terms	‘patient satisfaction’/exp. OR (patient* NEAR/2 satisfaction):ab,ti OR (patient* NEAR/2 reported):ab,ti OR ‘self report’/exp. OR (self NEAR/1 report*):ab,ti OR ‘patient preference’/exp. OR (patient* NEAR/2 preference*):ab,ti OR (patient* NEAR/1 assess*):ab,ti OR ‘self evaluation’:ab,ti OR ‘self evaluations’:ab,ti OR (patient* NEAR/2 rating):ab,ti OR (patient* NEAR/2 rated):ab,ti OR ‘self-completed’:ab,ti OR ‘self- administered’:ab,ti OR (self NEAR/1 assessment*):ab,ti OR ‘self-rated’:ab,ti OR ‘patient based outcome’:ab,ti OR ‘self evaluation’/exp. OR experience*:ab,ti
#4	ObsRO terms	‘observer reported’:ab,ti OR ‘observer rated’:ab,ti
#5	Population	‘population group’/exp. OR ‘population’/exp. OR population OR ‘age’/exp. OR age*:ab,ti OR ‘child’/exp. OR ‘adolescent’/exp. OR adolescent* OR child* OR teenage:ti OR kid:ti OR pediatr* OR neonatal:ab,ti OR ‘newborn’/exp. OR ‘infant’/exp. OR ‘preschool child’/exp.

### Data extraction

During the review process, eligibility assessment of both abstracts and full text articles were evaluated by two independent reviewers. In the case of non-agreement, a third senior reviewer made the final judgment. Relevant data were extracted from articles that were identified based on the inclusion/exclusion criteria and summarized in tables. For each article included in the review, an assessment on the quality of the data presented and therefore strength of results and recommendations was made. Each article was assigned a grade based on the type of article and strength of the data, as outlined by the following criteria:

A. Primary research; compares different response scales within the study.
B. Review or expert opinion; based on empirical evidence.
C. Primary research; evaluates a single response scale type within the study.
D. Review or expert opinion; based on expert consensus, convention or historical experience.

The letter grade A reflects the strongest empirical evidence for response scale recommendation and a letter grade D reflects the weakest empirically-based evidence.

## Results

**Fig. 1:**
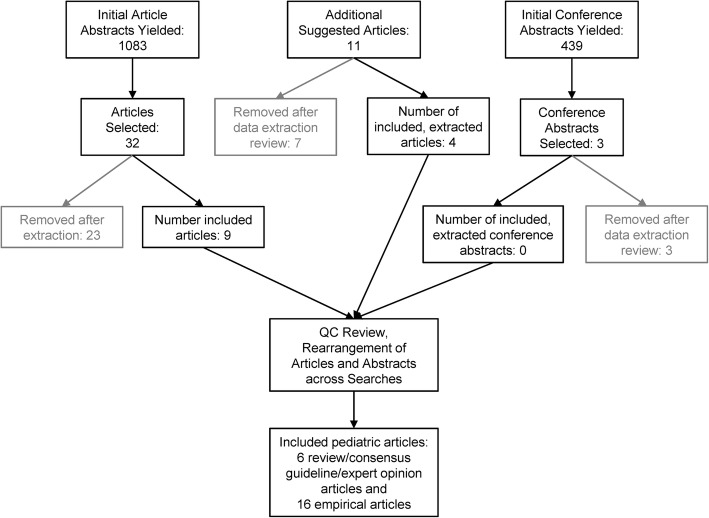
Screening and Review Process

The initial search yielded 1083 abstracts; a manual review of additional articles yielded 11 articles; lastly, a review of the potential 439 conference abstracts with the term “scale” yielded three potential abstracts. After abstract and full text screening and screening references from additional sources (full literature review results), in this review, we identified 6 review/consensus guideline/expert opinion articles and 16 empirical articles that provided guidance or evidence regarding pediatric response scale selection (Fig. 1). Three age groups (4 to 8 years, 6 to 18 years, and 10 to 18 years) emerged as most commonly described in the literature.

Across the review and expert opinion articles, there was general consensus that the 5-point VRS, including Likert scales, were appropriate for children 7 or 8 years of age and older (Table 2) [2, 6]. While graphical or faces scales are often used in pediatric studies [7, 8], some of the review articles noted that additional empirical evidence is needed to support the use of these scales and the specific ages for which they are appropriate [2]. There is some evidence that supports using a facial-graphics enhanced response scale in younger children (between the ages of 4 and 7) [9]. For children ages 7 or 8 and older, some review articles advocated use of NRS or VAS [9], whereas another article suggested that children prefer a VRS to an NRS or VAS [2]. Cohen et al. [10] noted that visual orientation of scales, emotions expressed in graphical scales, and word choice for verbal anchors can produce unexpected biases due to immaturity in abstract thinking skills of respondents; for example, a child might choose a numerical response based on favorite number rather than representation of experience.

**Table 2:** Summary of Key Evidence to Support Response Scale Selection for the Pediatric Population

Reference, Evidence Type^a^, and Grade^b^	Population	Response Option Type	Conclusions
Matza et al. 2004 [6], Direct, B	All children; referenced data supportive of children 8 years and older as well as younger children	Likert scales	Children 8 years and older have been shown to accurately use the full range of 5- and 7-point Likert scales to rate their health status, whereas children 7 and younger tend to use more extreme responses.
von Baeyer et al. 2006 [9], Direct, B	Children 5 years and older	Faces scales; Graphical scales; NRS; VAS; VRS	VRS may be appropriate for children 9 years and older as they require verbal fluency at a high level. NRS might be appropriate for children 8 years and older as they require numeracy and ability to think and express oneself in quantitative terms. VAS scales may be appropriate for children 6 years and older and have shown good psychometric properties. Faces scales may be appropriate for children 4 years and older as they may be simpler than equating one’s feelings to numbers of abstract verbal descriptors. Color scales allow children to select a color associated with level of pain and may be appropriate for children 4 years and older but have not been studied extensively. Pieces of hurt scales use 4 poker chips to represent amount of pain and may be appropriate for children 3 years and older.
Cohen et al. 2008 [10], Direct, B	Children 3 years and older	Faces scales; Graphical scales; VAS	Poker chip tool (children 3 to 4 years) VAS (children 8 years and older) Faces scales need to be considered and developed carefully given that affect portrayed (crying) might be taken literally and if the child is not crying, he/she might not select this option.
Tomlinson et al. 2010 [8], Direct, B	Children 5 years and older	Faces scales	For research use, the Faces scale has been recommended for children 5 years and older on the basis of utility and psychometric features.
D’Arcy 2011 [7], Direct, D	All children	Faces scales	The Faces scale is a reliable and valid tool that should be used in children of all ages.
Matza et al. 2013 [2], Direct, D	All children; referenced data supportive of children as young as 8 years old	Graphical scales; Likert scales; NRS;VAS	Studies have shown that children 8 years and older prefer Likert scale to VAS and 10-point NRS. Expert opinion believes pictorial illustrations to be helpful for children 7 years and younger but confirmatory research is needed.

^a^Direct evidence: Primary research that compares different response scales within study. Indirect evidence: Review or expert opinion based on empirical evidence

^b^Grade Key: A) Primary research; compares different response scales within the study; B) Review or expert opinion; based on an empirical evidence base; C) Primary research; evaluates a single response scale type within the study; and D) Review or expert opinion, based on expert consensus, convention, or historical experience

### Children between the ages of 4 and 8

Table 3 presents the empirical studies evaluating optimal response scale choice among the youngest of respondents (ranging in age from 4 up to 7 to 8). Liu et al. [5] found a 4-point VRS with graphical faces to aid in response-enhanced comprehension of the scale in children between 4 and 11 years of age. In a study evaluating various pain response scales for use in children between the ages of 6 and 8, results were inconclusive as to whether a Faces, NRS, VAS, or a color scale (e.g., children select a color associated with level of intensity), was superior [11]. These response scales did produce different estimates of pain and were not considered interchangeable in young children [11].

**Table 3:** Summary of key evidence to support response scale selection for children 4 to 8 years old

Reference, Evidence Type^a^, and Grade^b^	Article type/ population	Response Option Type	Conclusions
Liu et al. 2007 [5], Direct, C	Cross-Sectional Study; Children aged 4 to 11 years old	Faces scales; Likert-type VRS	In children 4 to 11 years, faces were combined with the Likert-type verbal categories to increase the children’s ability to understand the scale.
Sanchez-Rodriguez et al. 2012 [11], Direct, A	Cross-Sectional Study; Children aged 6 to 8 years old	Faces scales; Graphical scales; NRS; VAS	The Faces scale, NRS, Color scale, VAS cannot be used interchangeably to measure pediatric pain intensity. Of the four scales used, the NRS-11 produces the highest pain intensity score among children 6 to 8 years old while the VAS produces the lowest.

^a^Direct evidence: Primary research that compares different response scales within study. Indirect evidence: Review or expert opinion based on empirical evidence

^b^Grade Key: A) Primary research; compares different response scales within the study; B) Review or expert opinion; based on an empirical evidence base; C) Primary research; evaluates a single response scale type within the study; and D) Review or expert opinion, based on expert consensus, convention, or historical experience

### Children between the ages of 6 and 18

Table 4 includes the studies evaluating multiple response scales within the same study among children 6 years of age and older. Van Laerhoven et al. [12] found a 5-point VRS to be preferred over VAS or NRS in children 6 to 18 years of age, but all scale types showed comparable reliability. In contrast, Jylli et al. [13] found that younger children (ages 6 to 11 years) in their sample (overall range between 6 and 16 years of age) did not fully understand all descriptive words in a 5-point VRS, and that the VRS was not highly correlated with a pain VAS. Bailey et al. [14] found the VAS, NRS, and VRS to be reliable and valid in evaluating pain in children 8 to 17 years of age, but the scores produced were not interchangeable. Connelly and Neville [15] found that the VAS, NRS, and Faces scales tended to be highly inter-related, but scores were generally higher on the NRS, and that VAS and Faces were more responsive to decreasing trends in pain scores in children between the ages of 9 and 18. Bailey et al. [16] evaluated the correspondence between NRS, VAS, VAS with color, and Faces scales in children ages 8 to 18 who had abdominal pain, and found that, while there was high correspondence between the VAS and VAS with color, the NRS did not correspond with the other scale types. Benini et al. [17] found VAS to be easiest for children 7 to 18 years of age to understand and use as compared to Faces and other graphical scales, especially for children with mild or moderate developmental delay suffering from Down’s syndrome or spastic tetraplegia.

**Table 4:** Summary of key evidence to support response scale selection for children 6 to 18 years old

Reference, Evidence Type^a^, and Grade^b^	Article Type/ Population	Response Option Type	Conclusions
Benini et al. 2004 [17], Direct, A	Prospective Study; Children aged 7 to 18 years old	Graphical scales; VAS	In developmentally delayed children 7 to 18 years, the VAS scale was the easiest to use and understand, however, ratings were affected by emotions of participants (fear). The color scale was difficult for the children to understand, use and had difficulty interpreting Faces scales which includes an emotional overlay.
van Laerhoven et al. 2004 [12], Direct, A	Cross-Sectional Study; Children aged 6 to 18 years old	NRS; VAS; VRS	Children 6 to 18 years preferred the VRS over the NRS and VAS and find it easiest to complete. The VRS scale, the VAS and the NRS were of comparable reliability.
Jylli et al. 2006 [13], Direct, C	Cross-Sectional Study; Children aged 6 to 16 years old	VAS; VRS	Children 6 to 11 years knew fewer words than children 12 to 16 years to describe pain. Further studies are needed to determine the suitability of using 5-point VRS with word descriptors of pain with children of all ages. The study showed a weak correlation between the pain-rating index quotient for the sensory VRS and the VAS in the entire group.
Bailey et al. 2007 [16], Direct, A	Cross-Sectional Study; Children aged 8 to 18 years old	Graphical scales; NRS; VAS	Only the VAS and the VAS color analogue scale have acceptable agreement in children 8 to 18 years with moderate to severe acute abdominal pain. In particular, the NRS is not in agreement with the other evaluated scales and is not recommended for use in this population.
Bailey et al. 2010 [14], Direct, A	Prospective Study; Children aged 8 to 17 years old	NRS; VAS	The NRS provides a valid and reliable scale to evaluate acute pain in children aged 8–17 years but is not interchangeable with the VAS.
Connelly and Neville 2010 [15], Direct, A	Prospective Study; Children aged 9 to 18 years old	Faces scales; NRS; VAS	Results showed that in children 9 to 18 years, all 3 pain-intensity measures (Faces, VAS, and NRS) were highly interrelated, varied similarly with age and baseline state anxiety, and were comparably related to contemporaneous changes in affect. However, patients tended to rate pain intensity higher on the NRS, and the VAS and Faces Pain Scale-Revised were more responsive to decreasing trends in pain scores with elapsed surgical recovery time than the NRS.

^a^Direct evidence: Primary research that compares different response scales within study. Indirect evidence: Review or expert opinion based on empirical evidence

^b^Grade Key: A) Primary research; compares different response scales within the study; B) Review or expert opinion; based on an empirical evidence base; C) Primary research; evaluates a single response scale type within the study; and D) Review or expert opinion, based on expert consensus, convention or historical experience

### Children between the ages of 10 and 18

Table 5 presents studies evaluating response scale selection in older children (10 to 18 years). The NRS was found to produce higher scores than either the VAS or Faces scale, but the NRS had higher correspondence with the VAS than it had with Faces [18]. A 5-point VRS was found to be well accepted and understood [19]. Finally, a 5-point VRS was found to be stable regardless of recall period [20].

**Table 5:** Summary of key evidence to support response scale selection for children 10 to 18 years old

Reference, Evidence Type^a^, and Grade^b^	Article Type/ Population	Response Option Type	Conclusions
Lakkis et al. 2006 [19], Direct, C	Intervention Study; Children aged 10 to 15 years old	VRS	There were no difficulties with the completion of the questionnaires, suggesting that 5-point VRS can be administered effectively to children and adolescents aged 10 to 15 years. Study participants were able to discriminate between and respond to questions easily using the 5-point scale.
Takahashi & Yamamoto. 2006 [18], Direct, A	Prospective Study; Children aged 11 to 18 years old	Faces scales; NRS; VAS	In children 11 to 18 years, NRS produced higher scores than Faces or VAS but NRS and VAS had higher correspondence than with Faces.
Bennett et al. 2010 [20], Direct, C	Instrument Development and/or Validation study; Children aged 12 years and older	VRS	Two methods for measuring the 7-day symptom experience of patients 12 years and older with cystic fibrosis, in which the two methods (a single 7-day recall and repeated 24-h recall) were found to provide similar results for groups of patients using a 5-point VRS.

^a^Direct evidence: Primary research that compares different response scales within study. Indirect evidence: Review or expert opinion based on empirical evidence

^b^Grade Key: A) Primary research; compares different response scales within the study; B) Review or expert opinion; based on an empirical evidence base; C) Primary research; evaluates a single response scale type within the study; and D) Review or expert opinion, based on expert consensus, convention or historical experience

## Discussion

The aim of this review was to provide an overview of the published research on response scale option types used within pediatric populations. Results showed that there was empirical evidence supporting the use of VRS, NRS, and VAS response options in children and adolescents aged 8 to 18 years with age-appropriate literacy skills and cognitive development. There was also evidence that self-report instruments can be used for children as young as 4 years of age when graphical scales are used as the response scale option. Meanwhile, there was little support in the published literature for a preferred response scale option type in the age group 8 to 18 years. Our findings indicate the importance of evaluating different response options in cognitive interviews when developing a new or modifying an existing PRO measure.

In a 2007 review, Grange et al. stated that, for children younger than 5 years old, there was no clear empirical support for the use of self-report instruments [21]. Instead it was recommended that assessments regarding these children should rely upon clinical measures and observational reports. When they evaluated the psychometric properties of different health-related quality of life instruments that measure physical and/or emotional impact of symptoms, Grange et al. noted that these aspects may be just too abstract for children younger than 5 years old [21]. However, several studies have shown that children from the age of 4 years often can provide information on their health status, especially when it concerns concrete aspects, such as pain and use of medication [5, 11]. Well-defined, tangible concepts are important when considering self-report for this population.

When choosing response options to be used for 4 to 7 year olds, limitations in reading skills, vocabulary, and conceptual understanding of numbers must also be considered [2, 3]. Hence, the VRS, VAS, and NRS are likely not appropriate response options [2, 3, 9, 22]; whereas several instruments that use different graphical scales have shown acceptable validity and reliability (e.g., [5, 23]). However, there were aspects found among the acceptable graphical response options that were problematic. These included the expression of gender neutrality, the depiction of images resembling a target population or a stereotype of that population, and the recognition that the emotional cues expressed, such as smiling or frowning, in the faces in a graphical response option may be culturally dependent [5, 10].

As for the age group of 10 to 17 years, there was limited support in the published literature for any user-preferred response option. However, some studies have shown that the VAS appears to have shortcomings in this age group. Shields et al. [24] found that only one-third of study participants aged 5 to 14 were able to understand the VAS, and that those who were able to understand the VAS were significantly older (mean = 9.8 years) than those who did not understand it (mean = 8.2 years). More studies are needed to evaluate the robustness of these findings.

Development of PRO measures should be conducted so that the target population informs the selection of the type and the appropriate number of response options, recall period, attribution, etc. While VAS, NRS, VAS, and graphical scales have demonstrated to be reliable and valid formats when used in specific contexts among pediatric populations, response type used should be confirmed for each construct of interest and intended use [25]. The selection of responses should consider psychometric properties, represent the full continuum of the potential respondent experience, and should be ordered, equally spaced, and distinct from one another [4, 26, 27]. Ease of translatability and cultural adaptation should be considered and assessed early in development of PRO measures. While expert opinion and experience suggests the NRS may not pose problems in translation as the response numbers are not changed, the choice of word(s) used for NRS and VAS anchors may have implications for translations.

Typically 5- or 7-point scales are easier to translate than scales with more than seven response choices which can pose problems in other languages where more granular verbal distinctions do not exist. In a review of the translatability of various commonly used verbal anchors, agreement anchors (e.g., strongly agree, agree, disagree, strongly disagree) were found to be easy to translate and to have a high degree of equivalence across languages. In contrast, terms such as “fair” have multiple translation options and connotations across languages. Anchors such as “a little bit” are difficult to translate because of the lack of equivalence for the term “bit” in some languages. Further, item stems and response anchors corresponding to “…of the time” were found to be difficult to translate comparably across languages [28]. Authors recommended that the NRS or VAS should be used where possible, as those need only minimal translation [28].

Furthermore, the intended mode of data collection (e.g., paper/pen versus electronic) should be considered depending on the age of the person completing the instrument [29, 30]. Based on expert opinion and experience, an NRS response set can be easily implemented via electronic modality in an interactive voice response system, handheld (smartphone), tablet, and Web modes. However, formatting on handheld, tablet, and Web-based systems needs to be carefully considered so that the anchors of the NRS are associated with their intended number, and no ambiguity is caused by anchors that extend beyond one numerical category. It is impossible to implement a VAS using an interactive voice response system because the participant cannot place a mark on the line. Further, a VAS may be challenging to implement in other electronic modes due to screen size limitations and space constraints that cannot accommodate the 100-mm length presented on paper. Modifications such as a shorter line length that provides 101 data points can accomplish the same goal, as the electronic version scores the response automatically, eliminating the need for manual measurement using a ruler. For a VRS, the number of response options and length of verbal descriptors should be carefully selected so as to lighten the cognitive load (for an interactive voice response system) and to allow for equidistant formatting on one screen for handheld, tablet, and Web-based implementations. Faces scales, though less widely utilized in the PRO measurement literature reviewed, cannot be administered orally (via an interactive voice response system), but are easily administered via screen-based electronic modes.

This literature review was conducted in early 2015 and was limited to articles published in English during a 10-year timespan from 2004 through 2014, from which the key direct and indirect evidence was identified. Each article was graded based on the type of article and strength of the data. The search strategy was based on pre-specified criteria that may not have been inclusive of global research utilizing different terminology for PRO instruments designed for use in specific pediatric populations (e.g. “response format”, “response option”, “response set”, “item format”, “PROM”, or “patient-reported outcome(s)”), thus introducing risk of omitting relevant studies in the literature. Due to the scope of our literature review and the paucity of literature identified, more differentiated presentation of the findings was not pragmatic.

## Conclusion

The VRS, NRS, VAS, and graphical scales can all be reliable and valid response options in pediatric populations. However, the current empirical basis is insufficient to draw firm conclusions and to make differentiated recommendations. Therefore, when choosing a response format, it is important to consider the context of use during the development/modification of PRO measures and the study design. Apart from age, important aspects to consider are cultural background and cognitive development. More global studies on children’s preferences for response formats are needed to optimize pediatric self-reported measures. Additionally, more research is needed to assess the psychometric properties of items and their response options, to determine clinically meaningful changes and differences within pediatric clinical trials, which are impacted by the response scale chosen and the scoring function applied.

## Abbreviations

NRS: numeric rating scale
PRO: patient-reported outcome
VAS: visual analogue scale
VRS: verbal rating scale
